# Genotyping of *Capreolus pygargus* Fossil DNA from Denisova Cave Reveals Phylogenetic Relationships between Ancient and Modern Populations

**DOI:** 10.1371/journal.pone.0024045

**Published:** 2011-08-29

**Authors:** Nadezhda V. Vorobieva, Dmitry Y. Sherbakov, Anna S. Druzhkova, Roscoe Stanyon, Alexander A. Tsybankov, Sergey K. Vasil'ev, Mikhail V. Shunkov, Vladimir A. Trifonov, Alexander S. Graphodatsky

**Affiliations:** 1 Institute of Molecular and Cellular Biology of the Siberian Branch of the Russian Academy of Sciences, Novosibirsk, Russia; 2 Limnological Institute of the Siberian Branch of the Russian Academy of Sciences, Irkutsk, Russia; 3 Irkutsk State University, Irkutsk, Russia; 4 Department of Evolutionary Biology, University of Florence, Florence, Italy; 5 Institute of Archaeology and Ethnography of the Siberian Branch of the Russian Academy of Sciences, Novosibirsk, Russia; St. Petersburg Pasteur Institute, Russian Federation

## Abstract

**Background:**

The extant roe deer (*Capreolus* Gray, 1821) includes two species: the European roe deer (*C. capreolus*) and the Siberian roe deer (*C. pygargus*) that are distinguished by morphological and karyotypical differences. The Siberian roe deer occupies a vast area of Asia and is considerably less studied than the European roe deer. Modern systematics of the Siberian roe deer remain controversial with 4 morphological subspecies. Roe deer fossilized bones are quite abundant in Denisova cave (Altai Mountains, South Siberia), where dozens of both extant and extinct mammalian species from modern Holocene to Middle Pleistocene have been retrieved.

**Methodology/Principal Findings:**

We analyzed a 629 bp fragment of the mitochondrial control region from ancient bones of 10 Holocene and four Pleistocene Siberian roe deer from Denisova cave as well as 37 modern specimen belonging to populations from Altai, Tian Shan (Kyrgyzstan), Yakutia, Novosibirsk region and the Russian Far East. Genealogical reconstructions indicated that most Holocene haplotypes were probably ancestral for modern roe deer populations of Western Siberia and Tian Shan. One of the Pleistocene haplotypes was possibly ancestral for modern Yakutian populations, and two extinct Pleistocene haplotypes were close to modern roe deer from Tian Shan and Yakutia. Most modern geographical populations (except for West Siberian Plains) are heterogeneous and there is some tentative evidence for structure. However, we did not find any distinct phylogenetic signal characterizing particular subspecies in either modern or ancient samples.

**Conclusion/Significance:**

Analysis of mitochondrial DNA from both ancient and modern samples of Siberian roe deer shed new light on understanding the evolutionary history of roe deer. Our data indicate that during the last 50,000 years multiple replacements of populations of the Siberian roe deer took place in the Altai Mountains correlating with climatic changes. The Siberian roe deer represent a complex and heterogeneous species with high migration rates and without evident subspecies structure. Low genetic diversity of the West Siberian Plain population indicates a recent bottleneck or founder effect.

## Introduction

Denisova cave is located in the northwestern Altai Mountains on the right bank of the Anuy River. In spite of its small size the cave (with a central chamber and several short galleries) represents a unique source of Pleistocene deposits. The floor consists of 6-meter thick deposit with distinct lithological strata ranged from 280,000 BP to modern as dated by radiocarbon and RTL methods [1]. Recently the cave became well known due to the discovery of a previously unrecognized hominin species [2], [3].

Intensive excavations revealed fossils of 27 large mammalian species, both extinct and extant (wild horse, cave bear, cave hyena). Abundance of roe deer fossilized bones in strata dated from 50,000 years BP to present, prompted us to reconstruct the haplotypes of populations, which inhabited the Altai during Upper Pleistocene and Holocene and to compare them with modern populations.

The roe deer (*Capreolus* Gray, 1821) is one of the most widespread artiodactyl genera. It includes two species: the European roe deer (*C. capreolus*) and the Siberian roe deer (*C. pygargus*). The Siberian roe deer is considerably less studied than the European roe deer. In addition to moderate morphological differences between the species, the presence of B-chromosomes was shown to be a characteristic trait of the Siberian roe deer [4]. The genotyping of *C. pygargus* in two regions of Russia and north eastern China was reported [5]–[7], and two haplotypes of the Siberian roe deer from Korea were deposited on GenBank by Koh and Randi.

The modern systematics of the Siberian roe deer based mostly on morphological data is controversial, with most authors recognizing three subspecies. Some authors discriminate *C. p. pygargus*, inhabiting a large area from the Volga River to Lake Baikal and Yakutia, *C. p. tianschanicus* (or *C. c. bedfordi*, Thomas, 1908) spread in Tian Shan Mountains, Mongolia, Transbaikalia and Russian Far East, and *C. p. melanotis* (Miller, 1911) from the eastern Tibet and Chinese provinces Gansu and Sichuan [6], [8], [9]. Other authors do not consider *C. p. melanotis* as a separated subspecies [10], and are in agreement with Allen [11], by insisting on the identity of *C. p. melanotis* and *C. p. bedfordi*. According to Sokolov and Gromov [10] the subspecies *C. p. mantschuricus* is distributed across the Russian Far East, northern Korea and China. According to the last classification of Wilson and Reeder [12] the Siberian roe deer includes 4 subspecies: *C. p. bedfordi* (another name for *C. p. tianschanicus*), *C. p. mantschuricus*, *C. p. ochraceus* (from Korea) and *C. p. pygargus*.

Here we genotyped fossilized bones of 14 roe deer found in lithological strata 1–11 of the Denisova cave, thus covering the period from modern down to 50,000 years BP. We compared them to haplotypes of 37 modern Siberian roe deer from 5 populations of Altai, Tian Shan, Yakutia, Novosibirsk region and the Russian Far East.

## Materials and Methods

### Extraction of ancient DNA

Extraction of DNA was accomplished as described previously [13] with some modifications. The surface layer of the bone (0.5–1.0 mm) was removed with a drill: the rest was cut into 2–3 mm pieces and power grinded in a metal mortar. 1.0–1.5 g of powder was resuspended in 15 ml of 0.5 M EDTA pH 8.0, 0.5% N-lauryl sarcosyl (Sigma) and 0.5 mg/ml proteinase K. The suspension was then incubated at room temperature while mixing with a magnetic stirrer for 12–20 hours and then for 3 hours at 55°C. Undissolved pieces were removed by centrifuging at 5,000 g for 10 min. The supernatant was concentrated using Amicon Ultra-15 concentrator (Millipore) with an exclusion size of 5 Kd to a volume of 100–120 µl. The product was purified using the QIAquick PCR Purification Kit (Quiagen) according to the manufacturer's instruction. The resulted solution was aliquoted into 20 µl portions and stored at −20°C.

All manipulations with ancient DNA were accomplished using all the criteria of authenticity [14]–[16] in a separate sterile room, respecting all relevant measures to avoid contamination. All experiments included a negative control (solution without any bone material). Modern DNA samples were isolated in a separate room after all ancient DNA had been isolated.

The characteristics of ancient DNA samples and their accession numbers are given in Table 1.

**Table 1:** Characteristics of ancient samples of the Siberian roe deer.

№	samples	GenBankaccession	Archaeologicaldescription				Bones	haplotypes	identicalmodern
		№	layer	square	Depth,	Age,			samples
					m	thousand years		629 bp	629 bp
**Holocene**									
1	DC1	GU811824	def	E-2	0–0.5	0.3–1.8	rib	DC1	
2	DC2	GU811825	def	E-2	-//-	-//-	pipe bone	DC2	
3	DC3	GU811826	def	D-2	-//-	-//-	tooth	DC3	Ts1, Ts6, Ts7, Ts10, Ts13
4	DC4		2.1	D-2	-//-	1.8–2.7	pipe bone	DC1	
5	DC5	GU811827	2.3	E-3	-//-	-//-	tooth	DC5	Ns110
6	DC6	GU811828	2.3	D-3	-//-	-//-	tooth	DC6	Alt106, Ns99, Ns113, Ns108, Ns115
7	DC7	GU811829	2.3	D-4	-//-	-//-	tooth	DC7	
8	DC11		4.2	E-4	0.5–1.2	2.7–5.0	pipe bone	DC3	
9	DC12	GU811830	4.2	D-4	-//-	-//-	tooth	DC12	
10	DC13	GU811831	5	E-2	-//-	-//-	tooth	DC13	
**Pleistocеne**									
11	DC19	GU811833	9.3	D-2	1.55–1.65	21–24	phalanx	DC19	
12	DC17	GU811832	11.2	G-2	2.0–2.35	30–50	tooth	DC17	
13	DC24		11.2	E-2	-//-	-//-	pipe bone	DC17	
14	DC23	GU811834	11.3	G-4	-//-	-//-	instep bone	DC23	

### Modern Siberian roe deer sampling

The characteristics of the extant Siberian roe deer studied here are listed in Table 2.

**Table 2:** Characteristics of extant samples of the Siberian roe deer obtained in this study.

	samples	Accession №	region	haplotypes	Organ
	**Altai**				
1	Alt37	GU811820	Mountain Altai	Alt37	tissue culture
2	Alt40	GU811821	Mountain Altai	Alt40	tissue culture
3	Alt101	GU811822	Mountain Altai	Alt101	auricular cartilage
4	Alt102	GU811823	Mountain Altai	Alt102	auricular cartilage
5	Alt106		Mountain Altai	DC6	auricular cartilage
	**Yakutia**				
6	Ja96	GU811835	Khangalass	Ja96	tissue culture
7	Ja97	GU811836	Khangalass	Ja97	tissue culture
8	Ja105	GU811837	Khangalass	Ja105	auricular cartilage
9	Ja107		Khangalass	Ja96	auricular cartilage
10	Ja109		Khangalass	Ja96	auricular cartilage
11	Jg104	GU811838	Gornii	Jg104	auricular cartilage
	**Novosibirsk region**				
12	Ns99		Krasnoozerny	DC6	tissue culture
13	Ns108		Bolotninsky	DC6	auricular cartilage
14	Ns110		Zdvinsky	DC5	auricular cartilage
15	Ns111	GU811839	Zdvinsky	Ns111	auricular cartilage
16	Ns112		Chulimskiy	SIB1.1	auricular cartilage
17	Ns113		Chulimskiy	DC6	auricular cartilage
18	Ns114	GU811840	Karasuksky	Ns114	auricular cartilage
19	Ns115		Chulimskiy	DC6	auricular cartilage
20	Ns116	GU811841	Chulimskiy	Ns116	auricular cartilage
	**Tian Schan (Kyrgyzstan, Kyrgyzsky khrebet)**				
21	Ts1		Alamedin	DC3	tooth
22	Ts2	GU811842	Issik-Ata	Ts2	tooth
23	Ts3	GU811843	Issik-Ata	Ts3	tooth
24	Ts4	GU811844	Alamedin	Ts4	tooth
25	Ts5	GU811845	Issik-kul, Pokrovka	Ts5	tooth
26	Ts6		Issik-Ata	DC3	tooth
27	Ts7		Issik-Ata	DC3	tooth
28	Ts8		Alamedin	Ts5	tooth
29	Ts9		Chok-kurchak	SIB1.3	tooth
30	Ts10		Chok-kurchak	DC3	tooth
31	Ts11		Belogorka Sokuluk	Ts12	tooth
32	Ts12	GU811846	Dzhalomish	Ts12	tooth
33	Ts13		Chuiskaya obl.	DC3	tooth
	**Russian Far East**				
34	dv2078		Amur region	dv2078	liver
35	dv2079		-//-	dv2079	liver
36	dv2080		-//-	dv2079	liver
37	dv2083		-//-	dv2083	liver

**Figure 1:**
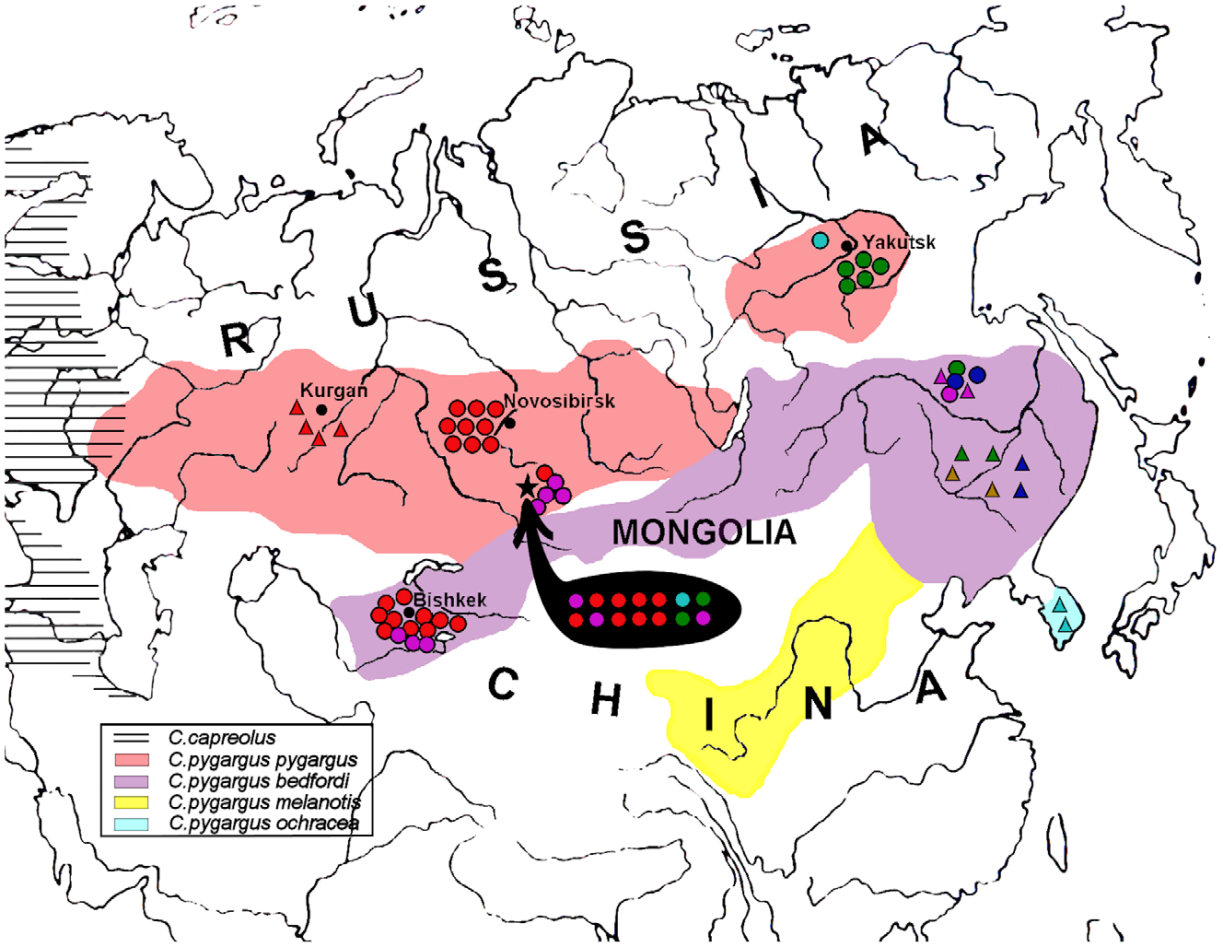
Geographical distribution of the samples superimposed on the modern distribution map of the Siberian and Europen roe deer [**27**]. Spheres represent the samples studied here first. The triangles indicate samples from literature [5], [7] and GenBank. The star indicates the localization of the Denisova cave. Ancient samples from Denisova Cave are indicated within the black area. The range of *C. p. melanotis* was described previously [9]. Blue – samples from cluster A, red – samples from cluster B, turquoise – samples from cluster C, green - samples from cluster D, violet - samples from cluster E, ochre - samples from cluster F. Ranges of separate subspecies are marked with color.

Figure 1 shows the location of samples we sequenced (circles) and those taken from previously published data [5], [7] and GenBank (triangles). Five tissue samples of the Siberian roe deer from Altai were provided by A. Sharshov, six tissue samples from Yakutia were provided by G.G. Boeskorov, four samples from the Russian Far East were given by I.V.Kartavtseva, 13 samples from Tian Shan were collected by A.G. Vorobiev and 9 samples from Novosibirsk region were provided by local hunters.

### DNA extraction

Ear cartilage tissue, 0.5–2.0 g, was chopped into pieces cooled with liquid nitrogen and crushed in a porcelain mortar. The powder obtained was suspended in 10–40 ml of buffer containing 0.01 M tris-HCl pH 8.0; 0.001 M EDTA, 0.2 mg/ml proteinase K and 0.2% SDS. The solution was incubated at 60°C for 2–3 hours and DNA was precipitated by adding NaCl to a final concentration of 0.5 M and an equal volume of ethanol. The pellet was washed twice in 70% ethanol, air dried and dissolved in 5 ml of TE buffer with 10 µg/ml RNAse A, followed by 3 hours of incubation at 37°C. DNA was ethanol precipitated, air dried and dissolved in 1 ml of TE buffer. The product was stored at −20°C.

### Amplification and sequencing

The primers for the mitochondrial control region of the Siberian roe deer were designed based on mitochondrial DNA sequence of *Bos taurus* and *Capreolus pygargus* from GenBank. Table 3 lists the primers used in this study covering up to 900 bp of the mitochondrial control region. The location of primers relative to Z70318 GenBank reference sequence is show in Figure S1.

**Table 3:** Primers used for amplification of both ancient and modern DNA.

			Tm	Positions in
name	sequence	length, nt	(100 mM NaCl)	Z70317.1
Cap1F	GCTGAAGTTCTATTTAAACTATTCCCTG	28	60,7	−28–0
Cap3F	AAAACCAAGAACTTTATCAGTATTAAA	27	55,9	24–50
Cap9F	ACAGCATAACTTAATGCGCTTATAGTAC	28	60,1	150–177
Cap11F	GTACATTATATTACATGCCCCATGC	25	60,4	214–238
Cap7F	CCTTGTCAACATGCGTATCCCG	22	65,9	321–342
Cap2F	CAAGGATCCCTCTTCTCGCTCC	22	65,0	398–419
Cap4F	GTGGGGGTAGCTATTTAATGAA	22	56,8	433–454
Cap6F	GGCATGGGCATGGCAGTCAA	20	68,4	704–723
Cap8F	CCTACAATTCTTTTCCCCCCCC	22	65,4	760–781
Cap9R	GTACTATAAGCGCATTAAGTTATGCTGT	28	60,4	150–177
Cap7R	GCATGGGGCATGTAATATAATGTAC	25	60,4	214–238
Cap3R	CGGCATGGTGATTAAGCTC	19	57,8	356–374
Cap1R	GGAGCGAGAAGAGGGATCC	19	59,8	400–419
Cap5R	GGGCGGTTTTAGGTGAGATGGC	22	67,4	485–506
Cap6R	TTGACTGCCATGCCCATGCC	20	68,4	704–723
Cap8R	GGGGGGGGAAAAGAATTGTAGG	22	65,4	760–781
Cap4R	AGTACTATTTGAGTATTGAAAATGCG	26	57,4	856–881
Cap2R	AATGGCGCTTAAATACTTACCTTGTCC	27	64,5	885–911

Amplification and sequencing of ancient DNA was accomplished using the whole set of primers. In the case of modern DNA two overlapping fragments were used, obtained with Cap1F/Cap5R and Cap2F/Cap2R primer pairs.

The 20 µl PCR reaction mixture contained 20 mM tris HCl pH 8.75, 10 mM KCl, 10 mM (NH_4_)_2_SO_4_, 0.1% Triton X-100, 2 mM MgCl_2_, 0.1 mg/ml nuclease-free BSA, 0.25 mM of each dNTP, 1 µM of each primer, 10–30 ng of DNA and 2.5 U of Pfu DNA polymerase. The PCR protocol included initial denaturing step at 95°C for 3 min, followed by 25–50 cycles of 94°C for 15 s, 60–62°C – 30 s and 72°C – 45 s.

Since all Pleistocene samples were significantly contaminated with bacterial and fungal DNA, we used two rounds of PCR amplification followed by gel electrophoresis, bands excision and DNA purification using the Gel Extraction Kit (QIAGEN), followed by PCR with internal primers.

The PCR product was purified using ExoSAP-IT (GE Healthcare) before the sequencing reaction. Sequencing was done in the Inter-institutional center of DNA sequencing SB RAS.

### Data analysis

The sequences were aligned using the CLUSTAL W version 1.8 software [17]. Haplotype and nucleotide diversity, transition/transversion parameter = 9.72, gamma distribution parameter alpha = 0.28, and the percentage of inter- and between populational variability was determined by Kimura 2p model (AMOVA; [18]) ARLEQUIN version 3.1 (http//cmpg.unibe.ch/software/arlequin3). The maximum likelihood (ML), maximum parsimony (MP), and neighbor-joining (NJ) methods were applied to construct the phylogenetic trees using PHYLIP package version 3.66 [19], likelihood and distance programs of MOBYLE PORTAL (http://mobyle.pasteur.fr/cgi-bin/portal.py). The sequence of the European roe deer (accession number in GenBank Z70318) was used as an outgroup. The gene genealogies between sequences were determined by statistical parsimony using TCS1.21 software [20].

Phylogenetic trees were inferred using Bayesian optimality criterion implemented in MrBayes v3.1.2 [21] and computed on the computer cluster. Models of nucleotide substitution were selected using an Akaike Information Criterion (AIC) in MrModeltest 2.2 [22]. The MCMC settings for each MrBayes analysis were: 2 runs, 10 chains each, for 2 million generations. Each MrBayes analysis was run three times independently to ensure that each run achieved similar stationary likelihood values (cold chain in stationary phase). Each run was considered to have reached a stationary distribution based on split frequencies reported in MrBayes and by plotting the log likelihood values of the cold chain. The MCMC runs were sampled every 100 generations, resulting in 20,000 trees per run. The first 5000 trees of each Bayesian run were discarded as burn-in, and the remaining trees in each analysis were used to calculate the posterior probabilities and 50% majority rule consensus tree.

## Results

### The ancient DNA: chronology and stratigraphy

Table 1 lists stratigraphic and chronological characteristics of ancient DNA samples obtained from the East Gallery, as well as similarity between the ancient and modern haplotypes. In total, 11 haplotypes were revealed in 14 samples. The sequences DC3/DC11 and DC17/DC24 looked identical on a short part of mitochondrial control region, but sequencing of an extended region showed that these samples belonged to different animals. Sequencing of 900 bp of DC1 and DC4 showed no difference between them. Further, the samples DC1, DC2 and DC3 were excavated from the deformed layer (a hole) from the stratum 2.2, where the bones of the same animal could be relocated to different positions. Although a post depositional mixing was reported for some area of the East Gallery [3], most of our samples were taken from clearly defined undisturbed strata.

The chronological characteristics of the profile using physical methods (radiocarbon, RTL, paleomagnetic) were assigned for the central, eastern and southern parts of the Denisova cave [1], [3], [23], [24], [25].

### Analysis of sequence and nucleotide distance

We obtained 629 bp long sequences of mitochondrial DNA control region for 14 ancient (11 haplotypes) and 37 modern (19 haplotypes) samples of the Siberian roe deer (Figure 1). The unique sequences were deposited in GenBank under accession numbers GU811824–GU811834 (ancient samples) and GU811820–GU811823, GU811835–GU811846 (modern samples). The correspondence between the sample names and GenBank numbers is shown in Tables 1 and 2. Position 1 of each sequence corresponds to the position 95 of the reference sequence Z70317 (mitochondrial sequence of the Siberian roe deer from GenBank). In addition, we integrated our data with all published haplotypes of the Siberian roe deer of a relevant length (Figure 1):

SIB1.1–SIB1.4 from Kurgan region [5];
Z70317 and SIB2.1 from the Amur region, Russian Far East [5];
SP1, WD1, WD2, WD3, WD4, XP1 (accession numbers AY854040–AY854045, respectively) from the north eastern China [7];
C.och1 and C.och2 (AJ311188 and AJ311189, respectively) from Korea (GenBank).

Table S1 lists the variable positions of all studied haplotypes: 62 polymorphic sites, disregarding indels and Table S2 shows substitution frequencies. Analysis of variable position distributions and nucleotide homology allowed us to determine the preliminary characteristics of the populations.

### Haplotypes of the ancient roe deer from the Denisova cave

Analysis of the variable sites (Table S1) and nucleotide distance (Table 4) showed a distinct separation of Holocene (DC1–DC13) and Pleistocene (DC17,19,23) haplotypes with considerable heterogeneity of Pleistocene haplotypes.

**Table 4:** Nucleotide distance of ancient haplotypes.

	DC1	DC2	DC3	DC5	DC6	DC7	DC12	DC13	DC17	DC19	DC23
DC1	**X**										
DC2	2,4	**X**									
DC3	2,4	**0,3**	**X**								
DC5	2,4	0,6	**0,3**	**X**							
DC6	2,8	0,6	**0,3**	**0,3**	**X**						
DC7	2,4	**0,3**	**0,3**	**0,3**	**0,3**	**X**					
DC12	2,3	0,8	**0,5**	**0,2**	**0,5**	**0,5**	**X**				
DC13	2,4	1.0	0.6	**0,3**	**0,3**	0,6	**0,5**	**X**			
DC17	2,6	1,8	1,8	1,5	1,8	1,5	1,6	1,8	**X**		
DC19	3,6	3,1	2,8	2,8	2,8	2,8	2,6	3,1	3,3	**X**	
DC23	3,4	2,1	2,1	2,4	2,4	2,1	2,6	2,8	3,3	2,6	**X**
Alt37	**0,3**	2,8	2,8	2,8	3,1	2,8	2,6	2,8	2,9	4.0	3,8
Alt40	2,6	2,3	2,3	2,3	2,6	2,3	2,4	2,6	2,8	4,1	**2,1**
Alt101	2,8	2,4	2,4	2,4	2,8	2,4	2,6	2,8	2,9	4,3	2,3
Alt102	**0,6**	3,1	3,1	3,1	3,4	3,1	2,9	3,1	3,3	4,3	4,1
Ns111	2,9	0,8	**0,5**	**0,5**	**0,2**	**0,5**	0,6	**0,5**	1,9	2,9	2,8
Ns114	2,1	0,6	**0,3**	**0,3**	0,6	0,6	**0,5**	0,6	1,8	3,1	2,4
Ns116	2,8	0,6	**0,3**	0,6	0,6	0,6	0,8	1.0	2,1	3,1	2,4
Ts2	2,6	**0,5**	**0,2**	**0,5**	**0,5**	**0,5**	0,6	0,8	1,9	2,9	2,3
Ts3	2,4	2,1	2,1	2,1	2,4	2,1	2,3	2,4	2,6	4.0	2,3
Ts4	2,6	0,8	**0,5**	**0,2**	**0,5**	**0,5**	**0,3**	**0,5**	1,6	2,9	2,6
Ts5	2,3	2,1	2,1	2,1	2,4	2,1	2,3	2,4	2,6	4.0	2,4
Ts12	2,9	0,8	**0,5**	**0,5**	**0,2**	**0,5**	0,8	**0,5**	1,9	2,9	2,3
Ja96	3,1	1,9	1,9	1,6	1,9	1,6	1,8	1,9	**0,5**	3,8	3,4
Ja97	3,1	2,3	2,3	1,9	2,3	1,9	2,1	2,3	0,8	4,1	3,8
Ja105	2,8	1,9	1,9	1,6	1,9	1,6	1,8	1,9	**0,2**	3,4	3,4
Jg104	2,4	1,9	1,5	1,3	1,6	1,6	1,1	1,6	2,1	**1,5**	3,1
SIB1.1*	2,3	**0,5**	**0,2**	**0,2**	**0,5**	**0,5**	**0,3**	**0,5**	1,6	2,9	2,3
SIB1.2*	2,6	**0,5**	**0,2**	**0,5**	**0,5**	**0,5**	0,6	0,8	1,9	2,9	2,3
SIB1.3*	2,6	**0,5**	**0,2**	**0,2**	**0,2**	**0,2**	**0,3**	**0,5**	1,6	2,6	2,3
SIB1.4*	2,6	1,1	0,8	0,8	1,1	1,1	1.0	1,1	2,3	3,6	2,9
SIB2.1*	2,6	2,3	1,9	1,9	2,3	2,3	2,1	2,3	2,8	3,8	2,4
Z70317*	2,4	2,1	1,8	1,8	2,1	2,1	1,9	2,1	2,6	3,6	2,3
dv2078	2,6	2,3	2,3	1,9	2,3	1,9	2,1	2,3	0,8	3,8	3,8
dv2079	2,6	2,1	2,1	1,8	2,1	1,8	1,6	2,1	1,6	3,3	3,3
dv2083	1,9	1,8	2,1	1,8	2,1	1,8	1,9	2,1	1,9	3,6	2,4
C.och1*	2,3	1,5	1,1	0,8	1,1	1,1	0,6	1,1	1,9	2,3	2,9
C.och2*	2,4	1,6	1,3	1.0	1,3	1,3	0,8	1,3	2,1	2,4	3,1
XP1*	2,8	1,6	1,6	1,3	1,6	1,3	1,5	1,6	**0,2**	3,5	3,1
SP1*	1,9	1,8	1,8	1,5	1,8	1,5	1,3	1,5	1,9	2,9	2,9
WD1*	2,3	1,1	1,1	0,8	1,1	0,8	1.0	1,1	**0,6**	2,9	2,6
WD2*	1,6	1,5	1,5	1,1	1,5	1,1	1.0	1,1	1,6	2,9	2,6
WD3*	2,4	1,9	1,9	1,6	1,9	1,6	1,4	1,9	1,4	3,1	3,1
WD4*	2,3	1,8	1,8	1,5	1,8	1,5	1,3	1,8	1,3	2,9	2,9
Z70318*	5,7	5,9	5,5	5,2	5,2	5,5	5.0	4,8	5,7	6,4	6,8

**Bold** are the minimal scores.

*haplotypes from GenBank and previously published data [5], [7].

All the Holocene haplotypes (excluding DC1) were very close to each other (0.2–0.5% nucleotide distance) and to modern Novosibirsk and Kurgan haplotypes (Table 4). It is noteworthy that DC5 was identical to modern Ns110; DC6 – to four modern Novosibirsk and one modern Altai samples, DC3 – to five modern Tian Shan samples.

The haplotype DC1 clustered together with modern Altai haplotypes Alt37 and Alt102 (nucleotide distance 0.3–0.6%) according to the characteristic variable sites distribution.

The Pleistocene haplotypes were all very different. DC17 was close to modern Yakutian haplotypes (0.2–0.5% nucleotide distance) and to Chinese XP1 haplotype (0.2% nucleotide distance). The haplotype DC19 was practically identical to the haplotype Jg109 from the Gornii region of Yakutia according to the characteristic distribution of the phylogenetically informative mutations in the first hypervariable region, although due to a large divergence in the rest of the sequence, the overall nucleotide distance was still large (1.5%).

The DC23 haplotype (as seen on Table 4) was rather distant from all modern haplotypes. The lowest nucleotide distance was found with Altai haplotype Alt40 (2.1%). The similarity in the nucleotide substitution pattern in the second domain of the control region (framed on the Table S1) in DC19 and DC 23 is noteworthy.

### The modern Altai population

The modern Altai population was found to be highly heterogeneous. Five representatives of the modern Altai population fall into three distinct groups: the first group has unique positions of variable sites and includes haplotypes Alt37, Alt102 as well as ancient DC1 (nucleotide distance 0.3–0.6%); the second group (haplotypes Alt40 and Alt101, nucleotide distance 0.2–0.3%) was closely related to haplotypes Ts3 and Ts5 from Tian Shan (nucleotide distance of the whole group 0.2–0.3%); sample Alt106 was identical to the ancient DC6 and comprises the third group.

The nucleotide distance between groups was 2.6–3.4%, whereas the nucleotide distance between the European and Siberian roe deer was 5.7–6.2%.

### The Yakutian population

The six samples studied revealed 4 haplotypes, with 3 haplotypes from the Khangalass region (Ja 96,97,105; nucleotide distance 0.3–1%) most closely related to Chinese XP1 (nucleotide distance 0.3–0.6%) and Pleistocene DC17 (0.2–0.8%), which was clearly ancestral to Ja105.

The only haplotype from the Gornii region (Jg104) was very divergent from the remaining Yakutian samples (nucleotide distance 2.3–2.9%), but was rather close to the two Korean, *C.pygargus ochracea*, haplotypes from GenBank (0.8 and 1%, respectively) and, as mentioned above, is related to the Pleistocene samples.

### The Tian Shan population

We detected 7 unique haplotypes in the 13 specimens of the Tian Shan population (see Table 2). Two haplogroups were quite distinct. The first one included Ts2, Ts4, Ts12 (the haplotype was represented by two identical samples Ts11 and Ts12) that were closely related to Novosibirsk and Kurgan haplotypes (nucleotide distance 0.2–0.6%); 5 samples (Ts1, Ts6, Ts7, Ts10, Ts13) were identical to the ancient DC3, and Ts9 was identical to Kurgan SIB1.3.

The second group includes haplotypes Ts3 and Ts5 (the latter was represented by two samples) that were very close to the haplotypes from the Russian Far East (SIB2.1 and Z70317), as well as to modern Altai haplotypes Alt40 and Alt101.

The distance between these two groups was 2.3%. The distance between the cluster that included these two groups and European roe deer was 5.7–5.9%.

### The Novosibirsk regional population

The Novosibirsk regional population was rather homogenous and was very close to published previously haplotypes from the Kurgan region [5]. The population was characterized by rather low nucleotide distance (0.2–0.8%) and was close to most Holocene haplotypes from the Denisova cave. Four of nine Novosibirsk samples were identical to ancient DC6, one to DC5, and one to SIB1.1.

### Russian Far East population

We analyzed 2 haplotypes published previously [5] - Z70317 and SIB2.1 (nucleotide distance 0.2%), and 4 samples (3 haplotypes - dv2078, dv2079, dv2083) first studied here. The haplotype dv2078 was very close (0.8%) to the ancient DC17, the haplotype dv2079 was similar to the Chinese haplotype WD4 [7] and dv2083 was close to both TS5 (0.6%) and Z70317 (0.8%).

### Population analysis

In spite of a relatively small sample size we made an estimate of the genetic diversity for each population, using both data obtained here and that previously published. The data, given in Table 5, show rather high haplotype diversity for all populations. The nucleotide distance within populations ranges from the lowest in the West Siberian Plain (0.0045) up to the highest in modern Altai population (0.0269). We attempted to determine the genetic relatedness from metric distances [18] but the result was inconclusive. This result could be explained by the fact that all populations except those from the West Siberian Plain had a high level of heterogeneity. Indeed, analysis of molecular variance (AMOVA) showed that the variance between populations (38%) was lower than the variance within populations (62%).

**Table 5:** Genetic diversity of the Siberian roe deer populations from different regions.

	Population	N	k	H(SD)	πSD)	V
1	Altai	5	5	1.0000 (0.1265)	0.0269 (0.0169)	28
2	Russian Far East	8	5	0.8929 (0.0858)	0.0182 (0.0105)	26
3	Tian Shan	13	7	0.8462 (0.0854)	0.0106 (0.0060)	16
4	Novosibirsk+KurganRegions*	14	9	0.9011 (0.0624)	0.0045 (0.0028)	9
5	Yakutia	6	4	0.8000 (0.1721)	0.0121 (0.0076)	19
6	China*	6	-	1.0000 (0.0962)	0.0113 (0.0071)	23
	total	52	36	0.9796 (0.0089)	0.0166 (0.0085)	61

N –the number of samples, k – the number of haplotypes, H – gene (haplotype) diversity, π - nucleotide diversity, V – the number of polymorphic sites, SD – standard deviation. Calculated according to Kimura model, the score of deletions, transitions and transversions equals to 1.

*- haplotypes from previously published data [5], [7].

### Phylogenetic and genealogical reconstructions

**Figure 2:**
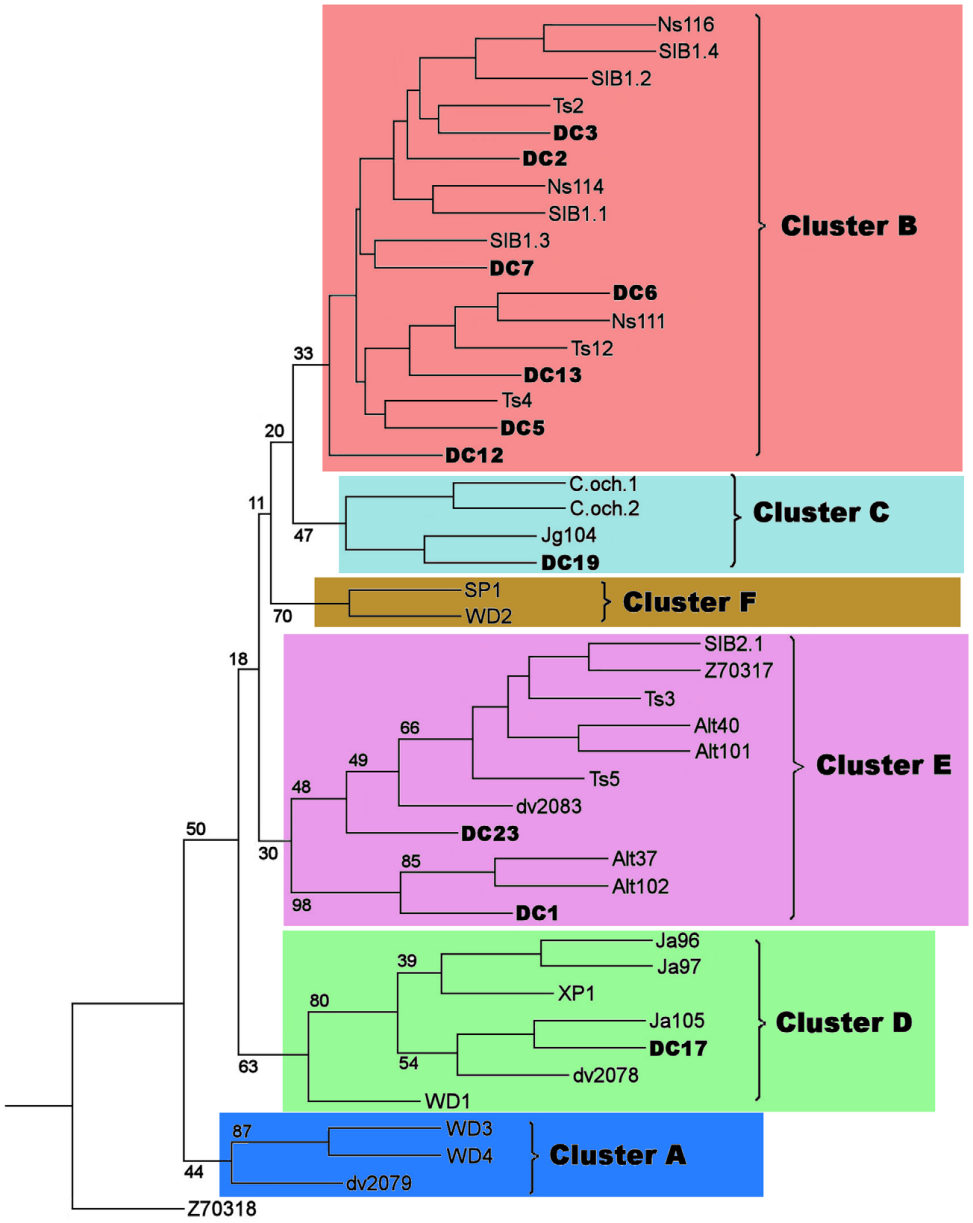
Maximum likelihood tree of Siberian roe deer mtDNA control region haplotypes with 100 bootstrapped replications. The transition/transversion parameter = 9.72 and gamma distribution parameter alpha = 0.28 were determined according to Kimura's two-parameter model (ARLEQUIN version 3.1). The major clusters and bootstrap values are indicated. The sequence of the European roe deer (Z70318) is taken as an outgroup. Abbreviations: “DC” indicate Denisova Cave samples, “Alt” – samples from Altai; “Ns” – Novosibirsk region samples; “Ts” – Tian Shan samples; “Ja” – Yakutian samples; “dv” – Russian Far East samples (studied here); “SIB2” and Z70317 - Russian Far East samples from [5]; “SIB1” – sample from Kurgan region [5]; “WD”, “XP”, “SP” – North Eastern China samples [7]; “C.och” – Korean samples (from GenBank). Clusters (A–F) are marked with different colors.

**Figure 3:**
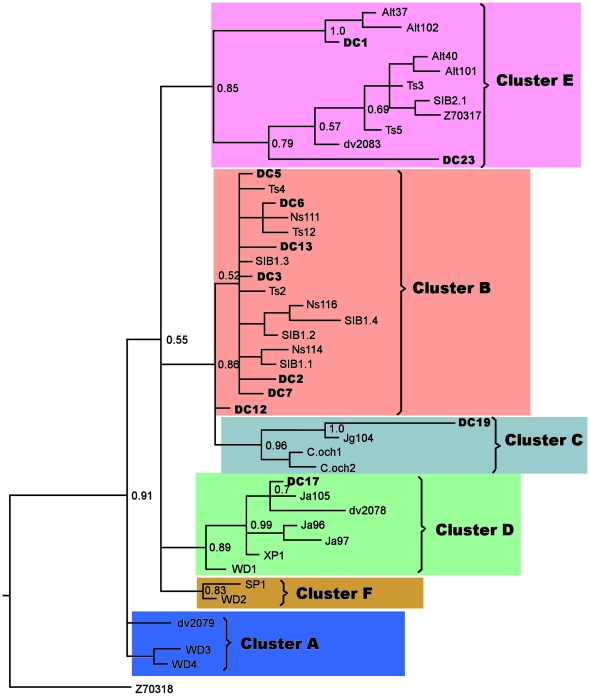
Phylogenetic tree inferred using Bayesian analysis of Siberian roe deer mtDNA control region haplotypes (the details of the method are described in the Data analysis capital, Materials and Methods). The sequence of the European roe deer (Z70318) is taken as an outgroup. Abbreviations: “DC” indicate Denisova Cave samples, “Alt” – samples from Altai; “Ns” – Novosibirsk region samples; “Ts” – Tian Shan samples; “Ja” – Yakutian samples; “dv” – Russian Far East samples (studied here); “SIB2” and Z70317 - Russian Far East samples from [5]; “SIB1” – sample from Kurgan region [5]; “WD”, “XP”, “SP” – North Eastern China samples [7]; “C.och” – Korean samples (from GenBank). Clusters (A–F) are marked with different colors.

**Figure 4:**
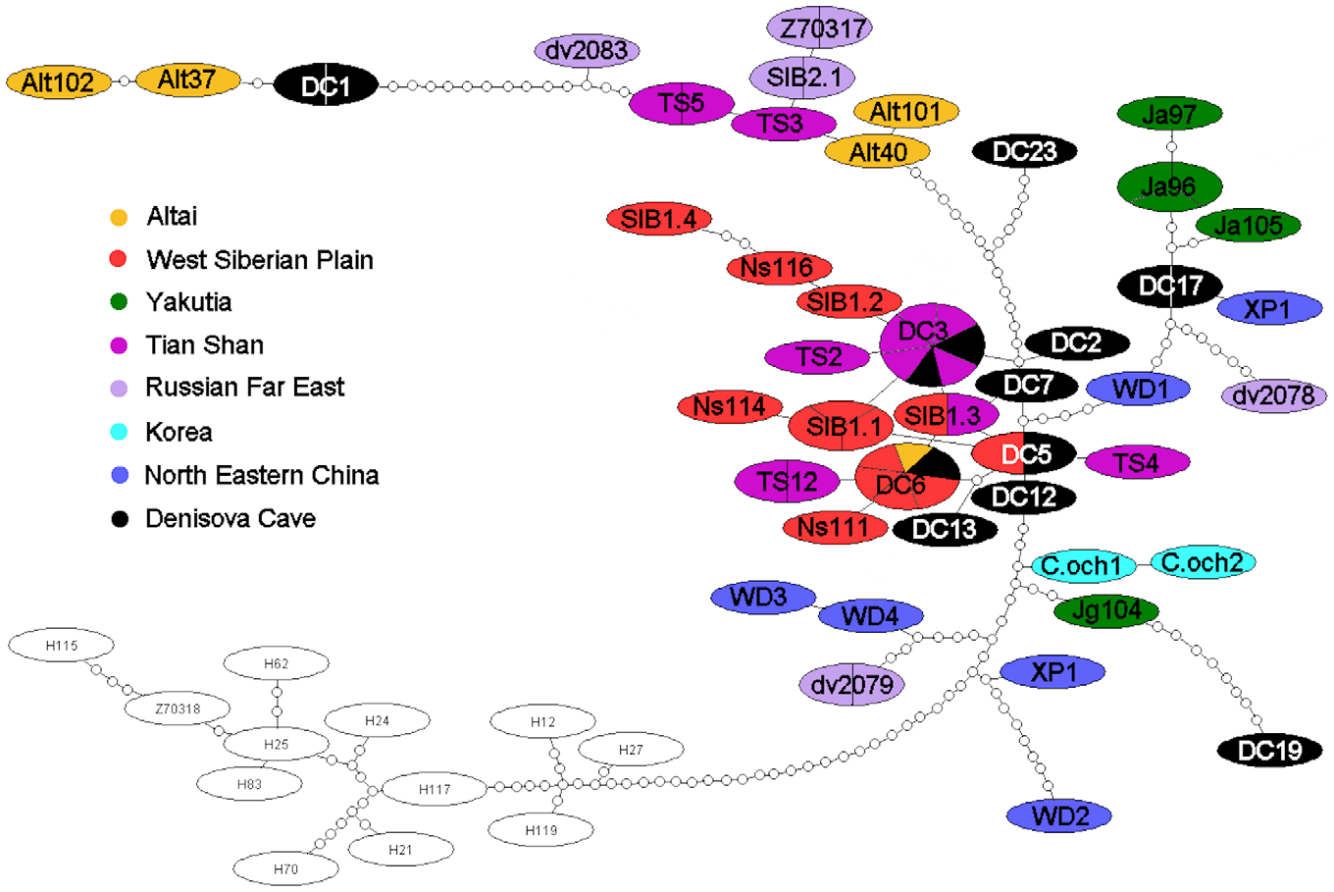
A cladogram of modern and ancient *C.pygargus* haplotypes obtained with TCS (v1.18) program. The size of the oval is proportional to the number of samples within each haplotype, each small circle on a branch indicates a mutation, including indels. Abbreviations: “DC” indicate Denisova Cave samples, “Alt” – samples from Altai; “Ns” – Novosibirsk region samples; “Ts” – Tian Shan samples; “Ja” – Yakutian samples; “dv” – Russian Far East samples (studied here); “SIB2” and Z70317 - Russian Far East samples [5]; “SIB1” – sample from Kurgan region [5]; “WD”, “XP”, “SP” – North Eastern China samples [7]; “C.och” – Korean samples (from GenBank). We took 12 Europen roe deer samples (empty ovals) representing all major branches of the phylogenetic tree constructed for the population of the European roe deer [26]. Color indicates the origin of samples.

Contemporary software for phylogenetic reconstructions failed to compare both modern and ancient haplotypes, because the programs were not able to place the sequence into the knot position. Moreover, the phylogenetic reconstructions of modern samples with the addition of ancient haplotypes always resulted in branch replacement and a decrease in bootstrap values. Therefore along with ML, NJ and MP methods we applied the Bayesian method as the most resistant to distortions. The Bayesian method was able to calculate the posterior probabilities of the branches, which provided statistical support for the topology. Further, it applied a 50% consensus, which left only the reliable knots, and collapsed the non-reliable branches into a soft polytomy. When using paleo-DNA sequences there is a high probability of bias in substitution frequencies due to DNA degradation, and the program should minimize these particular substitutions. Thus we used the J model test to select the appropriate model, which takes DNA degradation into account. We also applied the TCS-software [20], based on statistical parsimony, for the genetic genealogy reconstruction. Figure 2 shows the phylogenetic tree for 44 ancient and modern haplotypes of the Siberian roe deer, reconstructed by the maximal likelihood (ML) method with bootstrap support of 100%. A Bayesian phylogenetic tree is shown in Figure 3, and the TCS cladogram of all haplotypes is represented in Figure 4. The trees obtained with neighbor joining (NJ) and maximal parsimony (MP) methods are shown in Figure S2. The European roe deer sequence from GenBank was used as an outgroup.

Although the topology of some branches was poorly supported and collapsed into a polytomy on the Bayesian tree we still could trace some clusters (e.g. clusters A and D) that were rather invariable in content in most trees.

Cluster A included the northern Chinese haplotypes WD3 and WD4 that form a sister group on all phylogenies with a bootstrap value over 70%. Besides dv2079 from the Russian Far East is close to this group, as seen on TCS cladogram.

Cluster D (good supported on all trees) comprised all Yakutian haplotypes from the Khangalass region, the Pleistocene haplotype DC17, dv2078 (from the Russian Far East) and northern Chinese haplotypes WD1 and XP1. WD1 took a basal position within a cluster on all trees

The Korean haplotypes C.och1, C.och2 *(C. p. ochraceus)*, related to them Yakutian Jg104 (a haplotype from the Gornii region) and ancient DC19 formed cluster C (with a high Bayesian support). Cluster F contained only two haplotypes (SP1 and WD2) from the north-eastern China.

We divided the remaining poorly resolved haplotypes into two groups to allow a more wide ranging discussion: cluster B with all haplotypes from the Novosibirsk and Kurgan regions, some Tian Shan haplotypes as well as all Holocene haplotypes from Denisova cave except DC1 and cluster E with haplotypes from Tian Shan, Altai and Russian Far East.

To build the TCS cladogram we took 12 haplotypes of the European roe deer, representing all major branches of the phylogenetics tree, reconstructed previously for the whole European population [5].

The TCS cladogram mainly corroborated the data obtained from phylogenetic tree reconstruction. The Holocene haplotypes DC12, DC5, DC7 were ancestral for all modern West Siberian Plain roe deer and to most Tian Shan individuals, while DC1 was ancestral to some modern Altai roe deer. Among Pleistocene haplotypes, DC17, according to TCS cladogram, was ancestral to most modern Yakutian haplotypes, while DC19 was close to Yakutian Jg104 and related haplotypes from Korea.

The cladogram confirmed the close relationships between the West Siberian Plain and Yakutian populations, except for Jg104. However, it is noteworthy that the ancient haplotype related to Yakutian population (DC17) is 30,000 years older than the ancient haplotype related to West Siberian Plain population (DC12).

## Discussion

### Ancient DNA and authenticity of samples

It is well accepted that ancient DNA is better conserved in samples obtained from permafrost or from dry caves. Although the Denisova cave does not fit any of these criteria, we managed to isolate not only Holocene, but also Pleistocene DNA and we were able to genotype the ancient roe deer.

To ensure the authenticity of samples the following measures were undertaken: sample preparation, isolation of ancient DNA and setting up the reaction mixture for the first PCR round were accomplished in a special sterile box that was always free from modern DNA and any PCR products; isolation of modern DNA was done in a separate room and only after all experiments on ancient DNA sequencing were finished to exclude any possible contaminations; PCR products were either directly taken for sequencing (in case of pure bands on the gel) or were eluted from separate bands without product cloning. Thus we sequenced not separate clones, but the total product of same size sequences, what excludes the sequencing mistakes caused by aDNA degradation. Characteristic for chemical DNA degradation G→A and C→T substitutions occurred in Pleistocene samples with the same frequency as in modern samples, belonging to the same cluster (see Table S2).

Particularly noteworthy are Pleistocene haplotypes DC 19 and DC 23 that considerably differ form modern haplotypes by a high number of substitutions in the conserved domain. Figure S1 represents the scheme of DC23 sequencing. All sequences obtained had identical substitutions. Moreover, the absence of mixed peaks and high Q-value (55–61) in substitution positions exclude the possibility of mitochondrial nuclear insertion sequencing, since in this case we would inevitably observe a high number of mixed peaks and a high background.

### Phylogenetic trees

Low bootstrap values in phylogenetic trees reconstructed using mtDNA control region occur quite often in studies of both European and Siberian roe deer [6], [26]. We obtained phylohenetic trees with different algorithms – ML, NJ, MP and Bayesian (Figures 2, 3, Figure S2). Analysis of all trees topologies showed the there was almost no phylogenetic resolution. Although some elements of inner topology were supported by rather high bootstrap values, the relative positions of these branches were very poorly supported. The Bayesian tree was the most reliable, but most bootstrap values were low and all branches except clusters A and D were collapsed into polytomies, which prevented estimations of evolutionary relationships of different clusters. This was probably due to the high migration rates of the roe deer or a small dataset.

The analysis of nucleotide substitutions showed a relatively high divergence of the conserved domain in comparison to the variable domain. This difference might reflect a high level of reverse mutations and a restricted number of mutation sites in the hypervariable domain. It is evident that a high amount of recurrent mutations also hampered the phylogenetic analysis.

### Modern Siberian roe deer: taxonomical problems and polyphyletic populations

The taxonomy of various roe deer subspecies is controversial and based solely on morphometrical traits. Based on phylogenetic reconstructions and sequence analysis we can conclude that all populations studied here except for the West Siberian Plain population are highly heterogeneous, which is congruent with the long seasonal migration routes of roe deer and their ability to travel up to 100 km per day [27]. Indeed, there are not any natural or artificial barriers to prevent mixture of different populations of the Siberian roe deer on large sparsely populated area of Siberia, Kyrgyzstan and Russian Far East. Moreover, the Altai Mountains are located on the boundary between the presumed *C.p.pygargus* and *C.p.tianschaniscus* subspecies, while Russian Far East is known for migrations of both Manchurian and Yakutian roe deer (Figure 1).

Our results show the Siberian roe deer is highly variable and mobile species (see Figure 1), multiple, different haplogroups fall over all the proposed subspecies habitats and we did not detect any subspecies specific haplogroups.

### Dynamics of ancient roe deer populations in Altai and climatic fluctuations in Western Siberia

Paleontological, palinological and stratigraphic studies of the late Pleistocene in Western Siberia show that for the last 50,000 years significant climatic fluctuations caused drastic landscape changes. During the Kargin glaciation (33,000–30,000 years BP) and Sartan glacial maximum (20,000–18,000 years BP) large territories, now occupied by forests, were covered by tundra, which turned to forest-tundra in southern regions. Fauna of these periods included mammoth and other marker tundra animals – reindeer, lemming and polar fox [28]–[30]. Since Siberian roe deer prefers forests and woodlands, its range moved southwards together with wood ecosystems.

At the same time, climatic conditions of Altai Mountains were less dramatic. Palinological analysis of ancient deposits in Anuy valley indicates that during the Sartan period (20,000–10,000 years BP) the valley was occupied by broad-leaved forests that are now replaced by small leaved forests and taiga [1]. One hypothesis we considered was that Altai might have represented a refugial area (at least for forest species). Due its geographic features the Altai were apparently protected from active influence of ancient glaciations and secondly we found a relatively high genetic variability in this region.

Further we observed in this study a low genetic diversity of the West Siberian Plain roe deer, and it is probable that roe deer went almost extinct there. This data is corroborated by sparse roe deer fossil findings in Pleistocene excavations in the West Siberian Plain. During subsequent warming in Holocene roe deer repopulated northern plain regions. In contrast, we did not observe a significant reduction in haplotype diversity of Altai roe deer during the last 50,000 years and it can suggest that Altai roe deer never experienced extinction or even a bottleneck.

Nevertheless the analysis of ancient haplotypes form different layers could be interpreted to support an alternative hypothesis that the Altai was not a refugium of roe deer. First of all, the Pleistocene and Holocene populations are quite different from modern populations in the Altai, as well as from the populations of West Siberian Plain.

Genotyping of ancient DNA of the Siberian roe deer in Altai could be interpreted to support that over the last 50,000 years there were multiple replacements of roe deer populations, often correlated with climatic changes. Thus, in layer 11.3 dated as 50,000 years BP, the haplotype DC23 was found, which is related to modern roe deer of Tian Shan, north eastern China and the Russian Far East (see Figure 1). Layer 11.2 (with the upper dating 30,000 years BP) contained two roe deer samples (haplotype DC17), which are related to modern Yakutain haplotypes. DC19 haplotype (related haplotypes now distributed in Korea and Yakutia) was recovered from layer 9.3, containing deposits from the Konoshel cold phase (33,000–30,000 years BP). Holocene samples revealed new haplotypes DC2, 3, 5, 6, 7, 12 and 13 characteristic for modern West Siberian Plain populations. Finally, about 2,000 years BP a haplotype (DC1) very close to modern West Siberian Plain roe deer appeared again in Altai and occurs there up to present. Approximately at the same time the cold and wet Subatlantic climatic period began [31].

These data support the possibility that there were several replacements of the Altai population. If the alternative hypothesis is correct then the repopulation of large territories of the West Siberian Plains might have been made by roe deer that migrated from southern regions passing through Altai.

### Ancestral haplotypes and modern populations

TCS genealogy data (Figures 2,3 and 4) allow us to propose that DC12 could be ancestral to modern West Siberian Plain roe deer. TCS cladogram (Figure 4) clearly shows the genealogical sequence of DC12-DC5-DC7-DC3 haplotypes taking a basal position to both modern West Siberian Plain roe deer and some Tian Shan roe deer. Identity of five modern Tian Shan samples (Ts1,6,7,10,13) to the ancient Altai haplotype DC3 (see Table 1) and genealogical proximity of Ts2 and Ts12 haplotypes to ancient DC3 and DC6, respectively, assumes that 2–3 thousand years ago there might have been an expansion of Siberian populations in modern Kyrgyzstan.

According to TCS genealogy Pleistocene haplotype DC17 could be ancestral to most part of Yakutian roe deer population, since it is very close to modern Ja105 (only one substitution) and it takes a basal position to a branch including Ja95 and Ja96.

Haplotypes DC19 and DC23 (related to modern Korean and Tian Shan roe deer, respectively) are obviously extinct. A unique pattern of nucleotide substitutions in the conserved domain of CR never occurs in modern Siberian roe deer, whereas C→T (400) substitution (Table S1) is characteristic to most European roe deer. The haplotype DC19 related to modern Yakutian Jg104 and to geographically distant Korean roe deer *(C.p. ochraceus)* is particularly interesting as a “Korean trace” in Pleistocene Altai.

The lowest haplotype diversity (see Table 5) of the West Siberian Plain population could reflect founder or bottle neck effects. Unfortunately, samples dated to the glacial period (10,000–20,000 years BP), which would allow us to more precisely trace the chronology of roe deer expansion in western Siberia are not yet available.

### Conclusions

The results obtained in this study point to the uneven current distribution of haplotypes and dramatic evolutionary history of the Siberian roe deer populations. Analysis of a higher number of both ancient and modern samples from different locations and the use of larger number of independently evolving genomic regions (including nuclear DNA sequences) would help provide data needed to test various hypotheses and phylogenetic reconstructions presented here.

## Supporting Information

Figure S1 The scheme of DC23 sequencing. Black - forward sequences, red – reverse sequences. Vertical lines indicate the boundaries of 629 bp sequence used in this work. Position 1 of DC23 corresponds to position 95 of Z70317 (taken from GenBank). Top wine red bars represent substitution positions. Small arrows indicate positions of all primers from Table 3. Long arrows with small numbers indicate independent PCR reactions with different primer combinations: (1) - 1F/9R and 3F/9R (sequencing primer 9R); (2) – 3F/9R and 3F/7R (sequencing primer 7R); (3) – 1F/1R and 3F/3R (sequencing primers 3F and 3R); (4) – 11F/5R and 7F/5R (sequencing primer 5R); (5) – 2F/2R and 4F/8R (sequencing primer 8R); (6) – 2F/2R and 2F/4R (sequencing primers 2F and 4R).
(TIF)

Figure S2 Maximum parsimonious (A) and Neighbor-joining (B) trees of Siberian roe deer mtDNA control region haplotypes with 100 bootstrapped replications. The transition/transversion parameter = 9.72 and gamma distribution parameter alpha = 0.28 were determined according to Kimura's two-parameter model (ARLEQUIN version 3.1). The major clusters and bootstrap values are indicated. The sequence of the European roe deer (Z70318) is taken as an outgroup. Abbreviations: “DC” indicate Denisova Cave samples, “Alt” – samples from Altai; “Ns” – Novosibirsk region samples; “Ts” – Tian Shan samples; “Ja” – Yakutian samples; “dv” – Russian Far East samples (studied here); “SIB2” and Z70317 - Russian Far East samples [5]; “SIB1” – sample from Kurgan region [5]; “WD”, “XP”, “SP” – North Eastern China samples [7]; “C.och” – Korean samples (from GenBank). Clusters (A–F) are marked with different colors.
(TIF)

Table S1 Aligned sequences of variable sites of mtDNA CR (L-strand, 629 bp) for different populations of *Capreolus pygargus*. hapl – haplotype, pos – position of the substitution, bp – length of the sequence, N – the number of samples with the same haplotype, Cl - cluster. Ancient haplotypes are bold. Identical regions of DC19 and DC 23 are framed. Indels were excluded from the analysis. Nucleotide position 1 corresponds to 95 in Z70317 sequence. Nucleotide position 270 corresponds to the end of the first hypervariable domain [32].
(DOC)

Table S2 Substitution frequencies in CR mtDNA of different Siberian roe deer clusters.
(DOC)
