# Supplementary figures and images for: Genotyping of Capreolus pygargus Fossil DNA from Denisova Cave Reveals Phylogenetic Relationships between Ancient and Modern Populations
Source: PLoS One. 2011 Aug 29;6(8):e24045. doi: 10.1371/journal.pone.0024045 (PMC3163676; doi:10.1371/journal.pone.0024045)

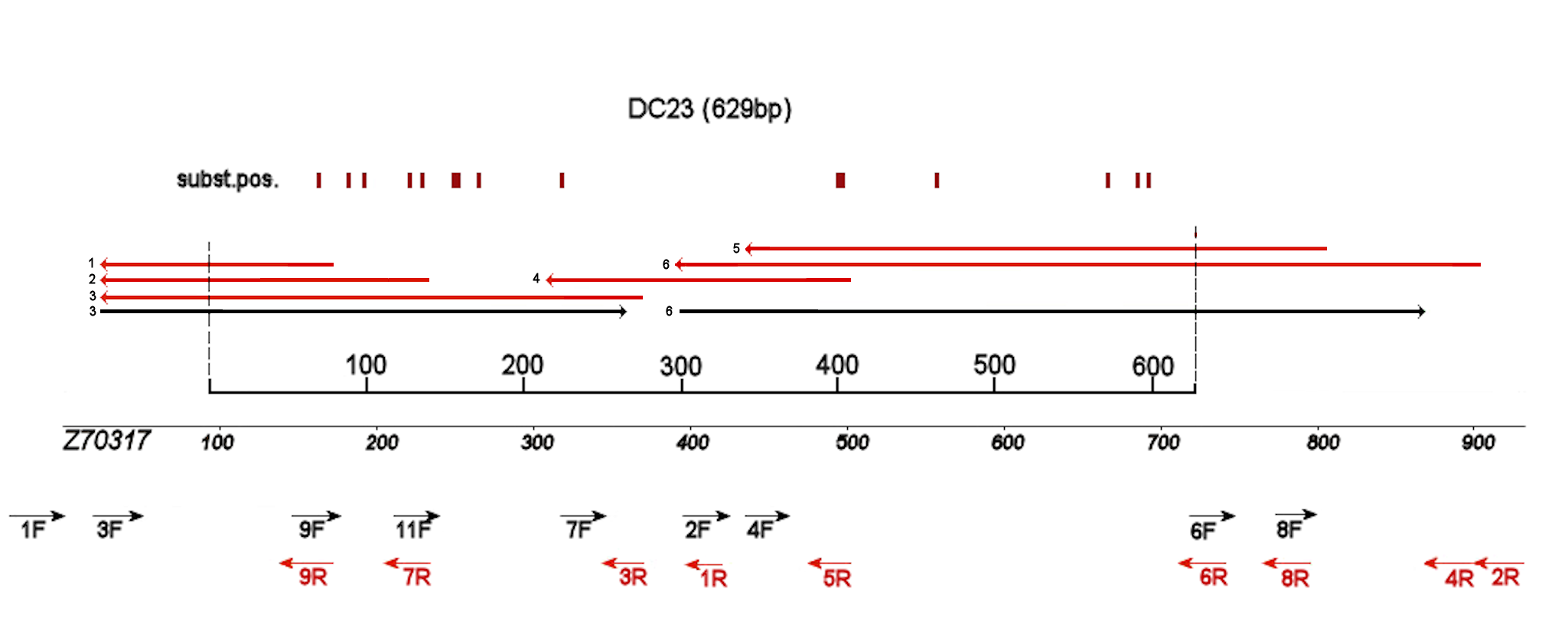

Supplement: Figure S1 — The scheme of DC23 sequencing. Black - forward sequences, red – reverse sequences. Vertical lines indicate the boundaries of 629 bp sequence used in this work. Position 1 of DC23 corresponds to position 95 of Z70317 (taken from GenBank). Top wine red bars represent substitution positions. Small arrows indicate positions of all primers from Table 3. Long arrows with small numbers indicate independent PCR reactions with different primer combinations: (1) - 1F/9R and 3F/9R (sequencing primer 9R); (2) – 3F/9R and 3F/7R (sequencing primer 7R); (3) – 1F/1R and 3F/3R (sequencing primers 3F and 3R); (4) – 11F/5R and 7F/5R (sequencing primer 5R); (5) – 2F/2R and 4F/8R (sequencing primer 8R); (6) – 2F/2R and 2F/4R (sequencing primers 2F and 4R). (TIF) [file pone.0024045.s001.tif]
