# Supplementary material for: Genotyping of Capreolus pygargus Fossil DNA from Denisova Cave Reveals Phylogenetic Relationships between Ancient and Modern Populations
Source: PLoS One. 2011 Aug 29;6(8):e24045. doi: 10.1371/journal.pone.0024045 (PMC3163676; doi:10.1371/journal.pone.0024045)
Supplement: Table S1 — Aligned sequences of variable sites of mtDNA CR (L-strand, 629 bp) for different populations of Capreolus pygargus. hapl – haplotype, pos – position of the substitution, bp – length of the sequence, N – the number of samples with the same haplotype, Cl - cluster. Ancient haplotypes are bold. Identical regions of DC19 and DC 23 are framed. Indels were excluded from the analysis. Nucleotide position 1 corresponds to 95 in Z70317 sequence. Nucleotide position 270 corresponds to the end of the first hypervariable domain [32]. (DOC) [file pone.0024045.s003.doc]

| hapl  pos | **DC6** | Ns111 | Ts12 | **DC13** | **DC7** | SIB1.3 | DC5 | Ts4 | **DC12** | Ns114 | SIB1.1 | Ts2 | **DC3** | **DC2** | Ns116 | SIB1.2 | SIB1.4 | | **DC17** | Ja105 | dv2078 | Ja96 | Ja97 | XP1 | WD1 | | Z70317 | SIB2.1 | Alt40 | Alt101 | **DC23** | Ts3 | Ts5 | dv2083 | **DC1** | Alt102 | Alt37 | | C.och1 | C.och2 | Jg104 | **DC19** | | SP1 | WD2 | | dv2079 | WD3 | WD4 | |
| --- | --- | --- | --- | --- | --- | --- | --- | --- | --- | --- | --- | --- | --- | --- | --- | --- | --- | --- | --- | --- | --- | --- | --- | --- | --- | --- | --- | --- | --- | --- | --- | --- | --- | --- | --- | --- | --- | --- | --- | --- | --- | --- | --- | --- | --- | --- | --- | --- | --- | --- |
| bp | **629** | . | . | **.** | **.** | . | . | . | **.** | . | . | . | **.** | **.** | . | . | . | | **.** | . | 631 | **.** | 631 | . | . | | . | . | . | . | **.** | . | . | . | **.** | **.** | . | | . | **.** | . | **.** | | **630** | 637 | | **.** | . | . | |
| N | **6** | 1 | 2 | **1** | **1** | 2 | 2 | 1 | **1** | 1 | 2 | 1 | **7** | **1** | 1 | 1 | 1 | | **2** | 1 | 1 | 3 | 1 | - | - | | 2 | 2 | 1 | 1 | **1** | 1 | 2 | 1 | **2** | 1 | 1 | | - | - | 1 | **1** | | - | - | | 2 | - | - | |
| 15 | **G** | . | . | **.** | **.** | . | . | . | **.** | . | . | . | **.** | **.** | . | . | . | | **.** | . | A | . | . | . | . | | . | . | . | . | **.** | . | . | . | **.** | . | . | | . | . | . | **.** | | . | . | | . | . | . | |
| 26 | **T** | . | . | **.** | **.** | . | . | . | **.** | . | . | . | **.** | **.** | . | . | . | | **.** | . | C | . | . | . | . | | . | . | . | . | **.** | . | . | . | **.** | . | . | | . | . | . | **.** | | . | . | | . | . | . | |
| 27 | **T** | . | . | **.** | **.** | . | . | . | **.** | . | . | . | **.** | **.** | . | . | . | | **.** | . | . | . | . | . | . | | . | . | . | . | **.** | . | . | . | **.** | . | . | | . | . | . | **.** | | . | . | | A | . | . | |
| 45 | **A** | . | . | **.** | **.** | . | . | . | **.** | . | . | . | **.** | **.** | G | G | G | | **.** | . | . | . | . | . | . | | . | . | . | . | **.** | . | . | . | **.** | . | . | | . | . | . | **.** | | . | . | | . | . | . | |
| 50 | **G** | . | . | **.** | **.** | . | . | . | **.** | . | . | . | **.** | **.** | . | . | . | | **.** | . | . | . | . | . | . | | . | . | . | . | **.** | . | . | . | **.** | . | . | | . | . | . | **.** | | . | . | | A | . | . | |
| 55 | **C** | . | . | **.** | **.** | . | . | . | **.** | . | . | . | **.** | **.** | . | . | . | | **.** | . | . | . | . | . | . | | . | . | . | . | **.** | . | . | . | **.** | T | T | | T | T | . | **.** | | . | . | | . | T | T | |
| 56 | **A** | . | . | **.** | **.** | . | . | . | **.** | . | . | . | **.** | **.** | . | . | . | | **.** | . | . | . | . | . | . | | . | . | . | . | **.** | . | . | . | **.** | . | . | | . | . | G | **G** | | . | . | | . | . | . | |
| 59 | **G** | . | . | **.** | **.** | . | . | . | **.** | . | . | . | **.** | **.** | . | . | . | | **.** | . | . | . | . | . | . | | A | A | A | A | **.** | A | A | . | **.** | . | . | | . | . | . | **.** | | . | . | | . | . | . | |
| 60 | **C** | . | . | **.** | **.** | . | . | . | **.** | . | . | . | **.** | **.** | . | . | . | | **.** | . | . | . | . | . | . | | . | . | . | . | **.** | . | . | . | **.** | . | . | | . | . | T | **T** | | . | . | | . | . | . | |
| 63 | **A** | . | . | **.** | **.** | . | . | . | **.** | . | . | . | **.** | **.** | . | . | . | | **.** | . | . | G | G | . | . | | . | . | . | . | **.** | . | . | . | **.** | . | . | | . | . | . | **.** | | . | . | | . | . | . | |
| 65 | **T** | . | . | **.** | **.** | . | . | . | **.** | . | . | . | **.** | **.** | . | . | . | | **C** | C | C | C | C | C | C | | C | C | C | C | **C** | C | C | C | **C** | C | C | | . | . | C | **C** | | C | C | | C | C | C | |
| 89 | **G** | . | . | **.** | **A** | . | . | . | **.** | . | . | . | **.** | **A** | . | . | . | | **A** | A | A | A | A | A | A | | . | . | A | A | **A** | A | A | A | **A** | A | A | | . | . | . | **.** | | A | A | | A | A | A | |
| 99 | **C** | . | . | **.** | **.** | . | . | . | **.** | . | . | . | **.** | **.** | . | . | . | | **.** | . | . | . | . | . | . | | T | T | T | T | **T** | T | T | T | **T** | T | T | | T | T | T | **T** | | T | T | | T | T | T | |
| 101 | **A** | . | . | **.** | **.** | . | . | . | **.** | G | . | . | **.** | **.** | . | . | . | | **.** | . | . | . | . | . | . | | . | . | . | . | **.** | . | . | . | **G** | G | G | | . | . | . | **.** | | . | . | | . | . | . | |
| 126 | **T** | . | . | **.** | **.** | . | . | . | **.** | . | . | . | **.** | **.** | . | . | . | | **.** | . | . | . | . | . | . | | . | . | C | C | **C** | . | . | . | **.** | . | . | | . | . | . | **.** | | . | . | | . | . | . | |
| 128 | **T** | . | . | **.** | **.** | . | . | . | **.** | . | . | . | **.** | **.** | . | . | . | | **.** | . | C | . | . | . | . | | . | . | . | . | **.** | . | . | . | **A** | A | A | | . | . | . | **.** | | . | . | | . | . | . | |
| 133 | **T** | . | . | **.** | **C** | C | C | C | **C** | C | C | C | **C** | **C** | C | C | C | | **C** | C | C | C | C | C | C | | C | C | C | C | **C** | C | C | C | **C** | C | C | | C | C | C | **C** | | C | C | | C | C | C | |
| 154 | **A** | . | . | **.** | **.** |  | . | . | **.** | G | G | G | **G** | **G** | G | G | G | | **.** | . | . | . | . | . | . | | G | G | G | G | **G** | G | G | G | **G** | G | G | | . | . | . | **.** | | . | . | | . | . | . | |
| 157 | **A** | . | . | **.** | **.** | . | . | . | **.** | . | . | . | **.** | **.** | . | . | . | | **.** | . | . | . | . | . | . | | T | T | T | T | **T** | T | C | C | **C** | C | C | | . | . | . | **.** | | . | . | | . | . | . | |
| 158 | **C** | . | . | **.** | **.** | . | . | T | **.** | . | . | . | **.** | **.** | . | . | . | | **.** | . | . | . | . | . | . | | . | . | . | . | **.** | . | . | . | **.** | . | . | | . | . | . | **.** | | . | . | | . | . | . | |
| 162 | **A** | . | . | **.** | **.** | . | . | . | **.** | . | . | . | **.** | **.** | . | . | . | | **.** | . | . | . | . | . | . | | . | . | . | . | **.** | . | . | . | **G** | G | G | | . | . | . | **.** | | . | . | | . | . | . | |
| 163 | **G** | . | . | **.** | **.** | . | . | . | **A** | . | . | . | **.** | **.** | . | . | . | | **.** | . | . | . | . | . | . | | . | . | . | . | **.** | . | . | . | **A** | A | A | | A | A | A | **A** | | A | A | | A | A | A | |
| 165 | **T** | . | . | **.** | **.** | . | . | . | **.** | . | . | . | **.** | **.** | . | . | . | | **.** | . | . | . | . | . | . | | . | . | . | . | **.** | . | . | . | **C** | C | C | | . | . | . | **.** | | . | . | | . | . | . | |
| 170 | **A** | . | . | **.** | **.** | . | . | . | **.** | . | . | . | **.** | **.** | . | . | . | | **.** | . | . | . | . | . | . | | G | G | G | G | **G** | G | G | G | **.** | . | . | | . | . | . | **.** | | . | . | | . | . | . | |
| 176 | **A** | . | . | **.** | **.** | . | . | . | **.** | . | . | . | **.** | **.** | . | . | . | | **G** | G | . | G | G | G | . | | . | . | . | . | **.** | . | . | . | **.** | . | . | | . | . | . | **.** | | . | . | | . | . | . | |
| 180 | **A** | . | . | **.** | **.** | . | . | . | **.** | . | . | . | **.** | **.** | . | . | . | | **G** | G | G | G | G | G | G | | . | . | . | . | **.** | . | . | . | **.** | . | . | | . | . | . | **.** | | . | . | | . | . | . | |
| 187 | **T** | . | . | **.** | **.** | . | . | . | **.** | . | . | . | **.** | **.** | . | . | . | | **C** | C | C | C | C | . | . | | . | . | . | . | **.** | . | . | . | **.** | . | . | | . | . | . | **.** | | . | . | | . | . | . | |
| 188 | **A** | . | . | **.** | **.** | . | . | . | **.** | . | . | . | **.** | **.** | . | . | . | | **.** | . | . | . | . | . | . | | . | . | . | . | **.** | . | . | . | **.** | . | . | | . | . | G | **G** | | . | . | | . | . | . | |
| 190 | **G** | . | . | **.** | **.** | . | . | . | **.** | . | . | . | **.** | **.** | . | . | . | | **A** | A | A | . | . | . | . | | . | . | . | . | **.** | . | . | . | **A** | A | A | | A | A | A | **A** | | . | . | | . | . | . | |
| 192 | **T** | . | . | **.** | **.** | . | . | . | **.** | . | . | . | **.** | **.** | . | . | . | | **.** | . | . | . | . | . | . | | . | . | . | . | **.** | . | . | . | **C** | C | C | |  |  | . | **.** | | . | . | | . | . | . | |
| 193 | **A** | . | . | **.** | **.** | . | . | . | **.** | . | . | . | **.** | **.** | . | . | . | | **.** | . | . | . | . | . | . | | . | . | . | . | **.** | . | . | . | **.** | . | . | | G | G | G | **G** | | G | . | | . | . | . | |
| 196 | **A** | . | . | **.** | **.** | . | . | . | **.** | . | . | . | **.** | **.** | . | . | . | | **G** | G | G | G | G | G | G | | . | . | . | . | **.** | . | . | G | **.** | . | . | | . | . | . | **.** | | . | . | | G | G | G | |
| 208 | **C** | . | . | **.** | **.** | . | . | . | **.** | . | . | . | **.** | **T** | . | . | . | | **.** | . | . | . | . | . | . | | . | . | . | . | **.** | . | . | . | **.** | . | . | | . | . | . | **.** | | . | . | | . | . | . | |
| 209 | **G** | . | . | **.** | **.** | . | . | . | **.** | . | . | . | **.** | **.** | . | . | . | | **.** | . | . | A | A | . | . | | . | . | . | . | **.** | . | . | . | **.** | . | . | | . | . | . | **.** | | . | . | | . | . | . | |
| 224 | **T** | . | C | **.** | **.** | . | . | . | **.** | . | . | . | **.** | **.** | . | . | . | | **.** | . | . | . | . | . | . | | C | C | C | C | **C** | C | C | . | **.** | . | . | | . | . | . | **.** | | . | . | | . | . | . | |
| 226 | **T** | . | . | **.** | **.** | . | . | . | **.** | . | . | . | **.** | **.** | . | . | . | | **.** | . | . | . | . | . | . | | . | . | . | . | **.** | . | . | . | **.** | C | C | | . | . | . | **.** | | . | . | | . | . | . | |
| 249 | **T** | . | . | **.** | **.** | . | . | . | **.** | . | . | . | **.** | **.** | . | . | . | | **C** | C | C | C | C | C | . | | . | . | . | . | **.** | . | . | . | **.** | . | . | | . | . | . | **.** | | C | C | | C | C | C | |
| 270 | **T** | . | . | **.** | **.** | . | . | . | **.** | . | . | . | **.** | **.** | . | . | . | | **.** | . | . | . | . | . | . | |  | . |  |  |  |  |  | . | **.** | . | . | | . | . | . | **.** | | . | . | | . | C | C | |
| 281 | **C** | . | . | **.** | **.** | . | . | . | **.** | . | . | . | **.** | **.** | . | . | . | | **.** | . | . | . | . | . | . | | . | . | . | . | **.** | . | . | . | **.** | . | . | | . | G | . | **.** | | . | . | | . | . | . | |
| 301 | **T** | . | . | **.** | **.** | . | . | . | **.** | . | . | . | **.** | **.** | . | . | . | | **C** | C | C | C | C | C | C | | C | C | C | C | **.** | C | C | C | **C** | C | C | | . | . | . | **C** | | . | . | | C | C | C | |
| 322 | **C** | . | . | **.** | **.** | . | . | . | **.** | . | . | . | **.** | **.** | . | . | . | | **.** | . | . | . | . | . | . | | . | . | . | . | **.** | . | . | . | **.** | G | . | | . | . | . | **.** | | . | . | | . | . | . | |
| 334 | **G** | . | . | **.** | **.** | . | . | . | **.** | . | . | . | **.** | **.** | . | . | . | | **.** | . | . | . | . | . | . | | . | . | . | . | **.** | . | . | . | **.** | . | . | | . | . | . | **.** | | . | . | | A | . | . | |
| 335 | **G** | . | . | **A** | **.** | . | A | A | **A** | A | A | . | **.** | **.** | . | . | A | | **A** | A | A | A | A | A | A | | A | A | A | A | **.** | A | A | A | **A** | A | A | | A | A | A | **.** | | A | A | | A | A | A | |
| 338 | **C** | . | . | **.** | **.** | . | . | . | **.** | . | . | . | **.** | **.** | . | . | T | | **.** | . | . | . | T | . | . | | T | T | T | T | **.** | T | T | T | **.** | T | . | | . | . | . | **.** | | . | . | | . | . | . | |
| 361 | **C** | . | . | **.** | **.** | . | . | . | **.** | . | . | . | **.** | **.** | . | . | T | | **.** | . | T | . | T | . | . | | T | T | T | T | **.** | T | T | T | **T** | T | T | | . | . | . | **.** | | T | T | | . | . | . | |
| 400 | **C** | . | . | **.** | **.** | . | . | . | **.** | . | . | . | **.** | **.** | . | . | . | | **.** | . | . | . | . | . | . | | . | . | . | . | **T** | . | . | . | **.** | . | . | | . | . | . | **T** | | . | . | | . | . | . | |
| 401 | **C** | . | . | **.** | **.** | . | . | . | **.** | . | . | . | **.** | **.** | . | . | . | | **.** | . | . | . | . | . | . | | . | . | . | . | **T** | . | . | . | **.** | . | . | | . | . | . | **T** | | . | . | | . | . | . | |
| 406 | **A** | . | . | **.** | **.** | . | . | . | **.** | . | . | . | **.** | **.** | . | . | . | | **.** | . | . | . | . | . | . | | . | . | . | C | **.** | . | . | . | **.** | . | . | | . | . | . | **.** | | . | . | | . | . | . | |
| 407 | **C** | . | . | **.** | **.** | . | . | . | **.** | . | . | T | **.** | **.** | . | . | . | | **.** | . | . | . | . | . | . | | T | T | T | T | **.** | T | T | T | **.** | . | . | | . | . | . | **.** | | T | . | | . | . | . | |
| 419 | **T** | . | . | **.** | **.** | . | . | . | **.** | . | . | . | **.** | **.** | . | . | . | | **.** | . | . | . | . | . | . | | . | C | C | C | **.** | C | C | . | **.** | . | . | | . | . | . | **.** | | . | . | | . | . | . | |
| 422 | **C** | . | . | **T** | **.** | . | . | . | **.** | . | . | . | **.** | **.** | . | . | . | | **.** | . | . | . | . | . | . | | . | . | . | . | **.** | . | . | . | **T** | T | T | | . | . | . | **.** | | T | T | | . | . | . | |
| 462 | **C** | . | . | **.** | **.** | . | . | . | **.** | . | . | . | **.** | **.** | . | . | . | | **.** | . | . | . | . | . | . | | . | . | . | . | **T** | . | . | . | **.** | . | . | | . | . | . | **T** | | . | . | | . | . | . | |
| 469 | **C** | G | . | **.** | **.** | . | . | . | **.** | . | . | . | **.** | **.** | . | . | . | | **.** | . | . | . | . | . | . | | . | . | . | . | **.** | . | . | . | **.** | . | . | | . | . | . | **.** | | . | . | | . | . | . | |
| 490 | **G** | . | . | **.** | **.** | . | . | . | **.** | . | . | . | **.** | **.** | A | . | A | | **.** | . | . | . | . | . | . | | . | . | . | . | **.** | . | . | . | **.** | . | . | | . | . | . | **.** | | . | . | | . | . | . | |
| 502 | **C** | . | . | **.** | **.** | . | . | . | **.** | . | . | . | **.** | **.** | . | . | . | | **.** | . | . | . | . | . | . | | . | . | . | . |  | . | . | . | **.** | . | . | | . | . | . | **.** | | . | . | | . | . | . | |
| 558 | **A** | . | . | **.** | **.** | . | . | . | **.** | . | . | . | **.** | **.** | . | . | . | | **.** | . | . | . | . | . | . | | . | . | . | . | **.** | . | . | . | **.** | . | . | | . | . | . | **G** | | . | . | | . | . | . | |
| 564 | **A** | . | . | **.** | **.** | . | . | . | **.** | . | . | . | **.** | **.** | . | . | . | | **.** | . | . | . | . | . | . | | . | . | . | . | **.** | . | . | . | **.** | . | . | | . | . | . | **G** | | . | . | | . | . | . | |
| 566 | **T** | . | . | **.** | **.** | . | . | . | **.** | . | . | . | **.** | **.** | . | . | . | | **.** | . | . | . | . | . | . | | . | . | . | . | **.** | . | . | . | **.** | . | . | | . | . | . | **.** | | . | . | | . | A | . | |
| 572 | **C** | . | . | **.** | **.** | . | . | . | **.** | . | . | . | **.** | **.** | . | . | . | | **.** | . | . | . | . | . | . | | . | . | . | . | **T** | . | . | . | **.** | . | . | | . | . | . | **T** | | . | . | | . | . | . | |
| 578 | **T** | . | . | **.** | **.** | . | . | . | **.** | . | . | . | **.** | **.** | . | . | . | | **.** | A | . | . | . | . | . | | . | . | . | . | **.** | . | . | . | **.** | . | . | | . | . | . | **.** | | . | . | | . | . | . | |
| 589 | **T** | . | . | **.** | **.** | . | . | . | **.** | . | . | . | **.** | **.** | . | . | . | | **.** | . | . | . | . | . | . | | . | . | . | . | **C** | . | . | . | **.** | . | . | | . | . | . | **.** | | . | . | | . | . | . | |
| 598 | **C** | . | . | **.** | **.** | . | . | . | **.** | . | . | . | **.** | **.** | . | . | . | | **.** | . | . | . | . | . | . | | . | . | . | . | **T** | . | . | . | **.** | . | . | | . | . | . | **T** | | . | . | | . | . | . | |
| Cl | B | | | | | | | | | | | | | | | | | D | | | | | | | | E | | | | | | | | | | | | **C** | | | | | F | | | A | | | |  |

Table S1. Aligned sequences of variable sites of mtDNA CR (L-strand, 629 bp) for different populations of Capreolus pygargus.

hapl – haplotype, pos – position of the substitution, bp – length of the sequence, N – the number of samples with the same haplotype, Cl - cluster. Ancient haplotypes are bold. Identical regions of DC19 and DC 23 are framed. Indels were excluded from the analysis. Nucleotide position 1 corresponds to 95 in Z70317 sequence. Nucleotide position 270 corresponds to the end of the first hypervariable domain [32].
