# Supplementary material for: Genotyping of Capreolus pygargus Fossil DNA from Denisova Cave Reveals Phylogenetic Relationships between Ancient and Modern Populations
Source: PLoS One. 2011 Aug 29;6(8):e24045. doi: 10.1371/journal.pone.0024045 (PMC3163676; doi:10.1371/journal.pone.0024045)
Supplement: Table S2 — Substitution frequencies in CR mtDNA of different Siberian roe deer clusters. (DOC) [file pone.0024045.s004.doc]

Table S2. Substitution frequencies in CR mtDNA of different Siberian roe deer clusters.

Hapl. – haplotypes (bold are ancient haplotypes), Sum 1 – the amount of G→A and C→T substitution, Sum 2- all rest substitutions, Clust. – clusters. Sum1/Sum2 – the ratio of G→A and C→T substitutions number to the rest substitutions number
